# The Effect of Fangcang Shelter Hospitals under Resource Constraints on the Spread of Epidemics

**DOI:** 10.3390/ijerph20105802

**Published:** 2023-05-12

**Authors:** Guangyu Li, Haifeng Du, Jiarui Fan, Xiaochen He, Wenhua Wang

**Affiliations:** School of Public Policy and Administration, Xi’an Jiaotong University, Xi’an 710049, China; uerlee@stu.xjtu.edu.cn (G.L.); haifengdu@mail.xjtu.edu.cn (H.D.);

**Keywords:** Fangcang shelter hospital, infectious disease model, medical resource allocation, epidemic prevention, resource constraints

## Abstract

Since the outbreak of the COVID-19 pandemic, Fangcang shelter hospitals have been built and operated in several cities, and have played a huge role in epidemic prevention and control. How to use medical resources effectively in order to maximize epidemic prevention and control is a big challenge that the government should address. In this paper, a two-stage infectious disease model was developed to analyze the role of Fangcang shelter hospitals in epidemic prevention and control, and examine the impact of medical resources allocation on epidemic prevention and control. Our model suggested that the Fangcang shelter hospital could effectively control the rapid spread of the epidemic, and for a very large city with a population of about 10 million and a relative shortage of medical resources, the model predicted that the final number of confirmed cases could be only 3.4% of the total population in the best case scenario. The paper further discusses the optimal solutions regarding medical resource allocation when medical resources are either limited or abundant. The results show that the optimal allocation ratio of resources between designated hospitals and Fangcang shelter hospitals varies with the amount of additional resources. When resources are relatively sufficient, the upper limit of the proportion of makeshift hospitals is about 91%, while the lower limit decreases with the increase in resources. Meanwhile, there is a negative correlation between the intensity of medical work and the proportion of distribution. Our work deepens our understanding of the role of Fangcang shelter hospitals in the pandemic and provides a reference for feasible strategies by which to contain the pandemic.

## 1. Introduction

Plagues such as the Spanish flu, Ebola and COVID-19 have had a huge impact on human economic production and daily life [1]. To rapidly control the spread of COVID-19, since the early days of the pandemic, Fangcang shelter hospitals have been constructed and placed into use in a number of cities. These facilities were formerly big venues such as sports arenas and conference centers [2]. Fangcang shelter hospitals serve to take care of patients with mild to moderate COVID-19 while providing basic medical care, frequent disease monitoring and rapid referral; they have been proven to be an effective public health facility to control the spread of the pandemic and have also been used in several other countries [3,4,5,6,7].

Infectious disease dynamics models are frequently used to quantify the effect of control strategies on transmission control [8]. They typically consist of a collection of equations for state changes that represent various chamber states and inter-chamber transitions. The transfer equation is a series of ordinary differential equations that describes how the number changes over time. The number of chambers indicates the population of that state. Flexible changes can be made to the chamber’s configuration to accommodate various research goals, as well as various infectious features [9,10], various control strategies [11,12], and resource limitations [13].

The propagation of infectious diseases is highly stochastic, according to past experience. Scholars have developed a range of infectious illness models to capture this stochastic process, such as adapting the infectious disease dynamics model to the random network [10,14] or including random processes in the transmission process. The fundamental unit of the network model is a distinct individual. The dynamics in these models are typically random or chance-driven processes because contagion occurs in diverse people in such models [15,16]. The stochastic process is used to randomize the deterministic propagation process of the dynamic model [17,18]. Additionally, some researchers have applied the fluid mechanics method to the infectious disease model and achieved some progress by using the chaotic aspects of fluid mechanics [19].

There are three areas of relevant research regarding Fangcang shelter hospitals. The first area focuses on introducing the basic situation of the cabin hospital [3,4,20]. The second area is mainly related to the use of the infectious disease model to quantitatively study the role of the hospital. Several infectious disease simulation studies have been conducted in order to examine the impact of Fangcang shelter hospitals from a variety of perspectives [6,7,21,22,23,24,25,26,27,28,29]. There has also been research conducted in order to study the physical and mental state of the medical staff and patients in Fangcang shelter hospitals [30,31]. In summary, Fangcang shelter hospitals are an important component complementing the existing medical system, and their role during health emergencies is one of the main research directions being addressed regarding medical system optimization [32,33,34,35,36]. However, the current research ignores the finite nature of medical resources and knowledge is still lacking regarding the effect of different medical resource allocation ratios between designated hospitals and Fangcang shelter hospitals on epidemic prevention and control. Furthermore, internal process optimization within Fangcang shelter hospitals has also not been addressed in previous studies. All these areas could be further studied in order to improve the efficiency and effectiveness of Fangcang shelter hospitals. As the recent COVID-19 mutant strain currently has a much higher transmission capacity and immunological escape capability than the original strain [37], although methods such as machine learning can help researchers quickly discover new potentially effective drugs [38], another wave of rapid and large-scale spread is still possible. Many countries are once again in danger of their healthcare systems being overwhelmed while facing a shortage of medical resources. In order to study the unique advantages of Fangcang shelter hospitals in treating mildly affected patients, we put forward the following questions: under the condition of insufficient extra medical resources, how effective are Fangcang shelter hospitals in preventing the spread of the epidemic? How should effective resources be allocated between designated hospitals and makeshift hospitals?

## 2. Model and Method

**Figure 1:**
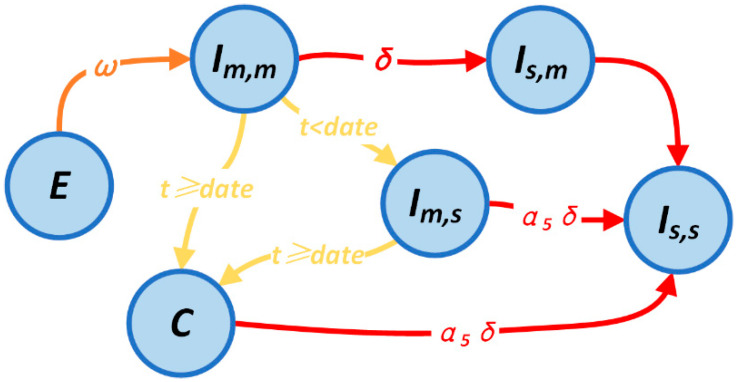
Schematic diagram of state transfer in confirmed patients. E denotes latent patients, $I_{m,m}$ denotes patients with mild disease at home, $I_{m,s}$ denotes patients with mild disease in hospital, $I_{s,m}$ denotes patients with severe disease at home, $I_{s,s}$ denotes patients with severe disease in hospital, and C denotes patients with mild disease in Fangcang shelter hospital.

To quantitatively answer this question, an infectious disease model with two stages based on the SEIR model was created in order to simulate the various scenarios of the epidemic prevention process [39]. Whether or not the Fangcang hospital is applied reflects the two stages of the model. We categorize the patients into five groups based on disease severity and whether they are receiving care at home or in a hospital. The state transfer procedure for confirmed patients is depicted in Figure 1.

In this model, both mildly and severely affected patients seek treatment at the designated hospital in the first stage as the Fangcang shelter hospital is not yet operational. The severe patients have priority access to the designated hospital, and both mild and severe patients are constrained by the limited amount of accommodation. Equation (1) shows the number of mild and severe patients who enter the designated hospital each day in the initial phase and are admitted in various compartments. T indicates the upper limit of the designated hospital, which we depict here by the number of hospital beds. The superscript denotes the number of individuals in the associated compartment after the iteration and before the transfer.

(1) $$\left\{\begin{array}{l} I_{m,m}=min\{max\{0,I-T\},I_{m,m}^{*}\}\\ I_{m,s}=max\{min\{I_{m,m}^{*}+I_{m,s}^{*},T-I_{s,m}^{*}-I_{s,s}^{*}\},I_{m,s}^{*}\}\\ I_{s,m}=max\{0,I_{m,s}^{*}+I_{s,m}^{*}+I_{s,s}^{*}-T\}\\ I_{s,s}=min\{I_{s,m}^{*}+I_{s,s}^{*},T-I_{m,s}^{*}\} \end{array}\right.$$

The second stage involves the opening of the Fangcang shelter hospital, where mild patients who are already being treated at the designated hospital are transferred; meanwhile, only severe patients are treated at the designated hospital, each with their own upper limitations. The number of mild patients entering various pods in the second stage is shown in Equation (2). $T^{\prime }$ denotes the new upper limit of the designated hospital and $T_{C}$ denotes the upper limit of the number of beds in the Fangcang shelter hospital. We also introduce the parameter date, which is not shown in the formula, but is very important. It is the dividing line between the first stage and the second stage, that is, the time when the Fangcang shelter hospital becomes operational.

(2) $$\left\{\begin{array}{c} I_{m,m}=max\{I_{m,m}^{*}+C^{*}-T_{C},0\}\\ C=min\{I_{m,m}^{*}+C^{*},T_{C}\}\\ I_{s,m}=max\{I_{s,m}^{*}+I_{s,s}^{*}-T^{\prime },0\}\\ I_{s,s}=min\{I_{s,m}^{*}+I_{s,s}^{*},T^{\prime }\} \end{array}\right.$$

**Figure 2:**
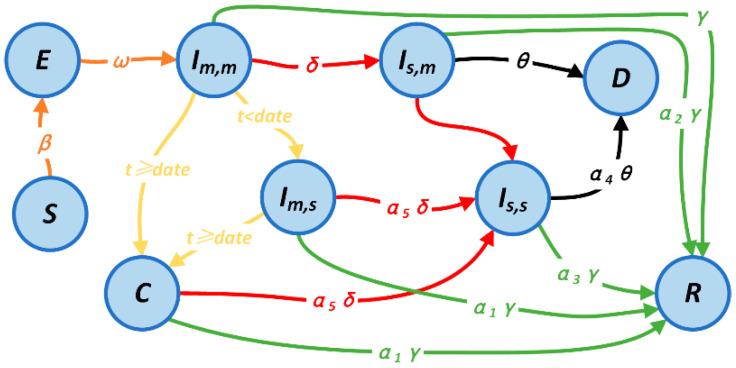
Schematic diagram of state transfer. S denotes susceptible, E denotes latent patients, $I_{m,m}$ denotes patients with mild disease at home, $I_{m,s}$ denotes patients with mild disease in hospital, $I_{s,m}$ denotes patients with severe disease at home, $I_{s,s}$ denotes patients with severe disease in hospital, and C denotes patients with mild disease in Fangcang shelter hospital. D denotes deceased patients, R denotes cured.

The estimate of the maximum capacity of the designated hospital and the Fangcang shelter hospital with new medical resources is shown in Equation (3). k denotes the total amount of medical resources input, which corresponds to the number of beds that can be assumed by the new medical resources in the designated hospitals. τ denotes the proportion of medical resources allocated, which indicates the proportion of medical resources invested in the Fangcang shelter hospital. η denotes the medical resource conversion rate, which corresponds to the ratio of the number of beds attended to by a group of healthcare workers in the Fangcang shelter hospital to those attended to in the designated hospital. The operational definitions of these three factors and their parameters, which explain the process of allocating medical resources, are presented in Supplementary material Table S1. In order to analyze the impact of the addition of the Fangcang shelter hospital’s on the death rate, we also introduced the death compartment *D*. The patient division and SEIR model were combined to create the final state transfer process, which is shown in Figure 2.

(3) $$\left\{\begin{array}{l} T^{\prime }=T+\left(1-\tau \right)k\\ T_{C}=\tau k\eta \end{array}\right.$$

Three categories of model parameters are employed in this study. Disease parameters, which are taken from traditional infectious disease transmission models and reflect the fundamental features of the disease, make up the first group [40,41,42]. The carrying capacity of different medical facilities in the city is reflected by the resource parameters, which include the maximum number of beds in designated hospitals, the quantity of new medical resources, and the maximum number of beds in Fangcang shelter hospitals. Medical factors, which take into account the severity of illness and various medical disorders, make up the third group of parameters. Based mostly on the researchers’ calculation, the model determines the adjustment coefficients that donate various medical conditions and sickness severities to the chances of recovery or death based on the recovery rates of mild patients and the mortality rates of severe patients, respectively.

**Figure 3:**
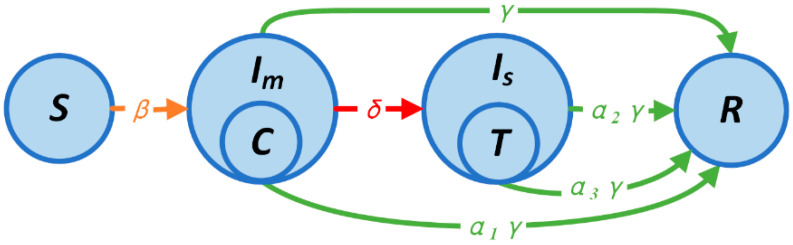
Schematic diagram of simple model transfer. *C* and *T* represent Fangcang shelter hospitals and designated hospitals, and $I_{m}$ and $I_{s}$ signify mild patients and severe patients, respectively.

We presented a one-stage simple model that significantly simplifies the two-stage model to aid in the analytical analysis. The transfer procedure of the model is shown in Figure 3. We eliminated the incubation process and just preserved the most crucial states because the SEIR model exhibited asymptotic behavior that was nearly identical to that of the SIR model [43]. The worst-case scenario is if the outbreak has already started and has gone beyond the maximum capacity of the designated and Fangcang hospitals, making it impossible to treat even a small proportion of mild and severely affected patients. The behavior of some Fangcang shelter hospitals in Wuhan illustrates the viability of this strong premise. It is also assumed that mild patients in Fang shelter hospitals can entirely avoid becoming severe patients. Because of the high patient volume, Fangcang shelter hospitals and designated hospitals are directly expressed as the highest limits of both. Equation (4) depicts the state transfer process, and the parameters’ significance is the same as that of the two-stage model’s parameters, where β, γ, and δ quantify the transmission rate to the susceptible status, severity rate, and recover rate, respectively. Additionally, T and C denote the carrying capacity of the designated and Fangcang hospitals, and $\alpha_{1}$, $\alpha_{2}$, and $\alpha_{3}$ represent the correction factor of the recovery rate for mild patients at home, severe patients at home, and severe patients in the designated hospital, respectively.

(4) $$\left\{\begin{array}{l} \dot{S}=-\beta S\left(I_{m}-C\right)-\beta S\left(I_{s}-T\right)\\ \dot{I_{m}}=\beta S\left(I_{m}-C\right)+\beta S\left(I_{s}-T\right)-\alpha_{1}\gamma C-\gamma \left(I_{m}-C\right)-\delta \left(I_{m}-C\right)\\ \dot{I_{s}}=\delta \left(I_{m}-C\right)-\alpha_{3}\gamma T-\alpha_{2}\gamma \left(I_{s}-T\right)\\ \dot{R}=\alpha_{1}\gamma C+\gamma \left(I_{m}-C\right)+\alpha_{3}\gamma T+\alpha_{2}\gamma \left(I_{s}-T\right) \end{array}\right.$$

The requirement that must be met denotes the extent to which the number of patients increases with the number of susceptible patients at a given level of prevalence; this can be obtained by setting the derivative of the number of confirmed patients. When this requirement is met, the epidemic hits an inflection point and the number of current patients stops growing. This requirement can be expressed as $\frac{dI}{dS}=0$, which means that the number of patients no longer varies with the number of susceptible patients, which is actually an inflection point that represents a continuous decline in the number of current patients in the future until the end. We can simplify the equation, and if we ignore the very small a and b, we obtain Equation (5), where $I_{0}$ denotes the number of patients at the time the Fangcang hospital is put into operation, $T_{0}$ denotes the number of beds at the very beginning of the designated hospital, and $R_{0}$ denotes the basic number of infections during the epidemic. To simplify the equation, x is actually equal to $\frac{\beta S_{0}}{\gamma }$. We borrow here the conclusions from the classical SIR model, and since there is no difference in the final trend between the SIR and SEIR models, we believe that such borrowing is reasonable if used only for qualitative analysis. For a particular time moment, the right-hand side of the equation is an expression that is entirely determined by constants that do not change in the short term, such as the properties of the virus, the number of local medical resources, and the condition of local medical resources; meanwhile, in the left-hand side of the equation, the two parameters we are concerned with are multiplicative, that is to say, under particular resource conditions, this is the ideal allocation ratio, and the conversion rate follows the inverse proportional relationship. The number of patients infected at the top of the curve is much smaller than that at the bottom. It should be noted that the removal of the state of latent patients will result in a lower allocation ratio than that of the simulation when derived using Equation (5). The variation relationship implied by this equation will be confirmed in the following section of this paper.

(5) $$\tau \left[R_{0}\left(\eta -1\right)+\alpha_{1}\eta -\eta \right]=\frac{R_{0}(I_{0}-T_{0}-k)-I_{m}}{k}$$

We conduct simulations to determine the effectiveness and size of the role of Fangcang shelter hospitals in epidemic prevention and control with different allocations and efficiencies. These simulations will be used to determine the number of uninfected individuals who remain after the Fangcang shelter hospitals are put into use.

(i): The Fangcang shelter hospitals’ efficacy. The effectiveness of the medical resources invested in the Fangcang shelter hospitals in order to prevent the epidemic’s spread is examined in this section. In addition, the connection between the quantity of emergency resources built and the epidemic’s spread when those resources are used to build Fangcang shelter hospitals are also examined. Specifically, we set τ=0.95, η=10, date=10 to observe the change in the proportion of the susceptible population over time after the construction of the Fangcang shelter hospital. To confirm that the model could accurately depict the ability of Fangcang shelter hospitals to slow the spread of the epidemic, the number of afflicted individuals with regard to various numbers of constructed Fangcang shelter hospitals was considered. The Fangcang shelter hospitals serve as an addition to the designated hospitals by isolating a large number of mild patients, thus lowering the risk that mild patients who are isolated at home, owing to a shortage of medical resources, pose to other susceptible people. After the Fangcang shelter hospitals start operating, we anticipate that the rate of vulnerable patients becoming infected will drop significantly, and that the proportion of susceptible patients who are still alive will climb as the number of Fangcang shelter hospitals increases.

(ii): Impact of the use and distribution of medical resources. This section investigates whether changing medical resource allocation and use have an effect on stopping the epidemic’s spread. Specifically, we set date=10, and observe the changes in the proportion of remaining susceptible people after the epidemic stabilizes under different conversion rates or allocation ratios. It was expected that the proportion of still-infected people would keep rising as the conversion ratio increased, indicating that increasing the conversion ratio could help contain the epidemic spread. The proportion of susceptible people under different conversion ratios demonstrated that improving the Fangcang shelter hospital’s workflow could help to contain the epidemic and prevent spread. The marginal changes in the proportion of susceptible individuals and the proportion of susceptible individuals with the change in the distribution ratio at different distribution ratios are proof that the distribution ratio represented by Equation (5) satisfies the relationship. It is expected that the number of remaining susceptible individuals will rise and then fall as the distribution ratio increases.

(iii): Optimal conversion rate and allocation ratio combinations. When the additional medical resources are constant, this section investigates the effects of various combinations of allocation ratios and conversion rates on containing the epidemic’s spread. We specifically set k=1000 and date=10, and monitor the proportion of the susceptible population that is still present once the outbreak has settled using various combinations of conversion and allocation ratios. The best combination region is determined by comparing the results of various combinations, and we anticipate that the optimal combination zone will be the region that complies with Equation (5).

## 3. Results

**Figure 4:**
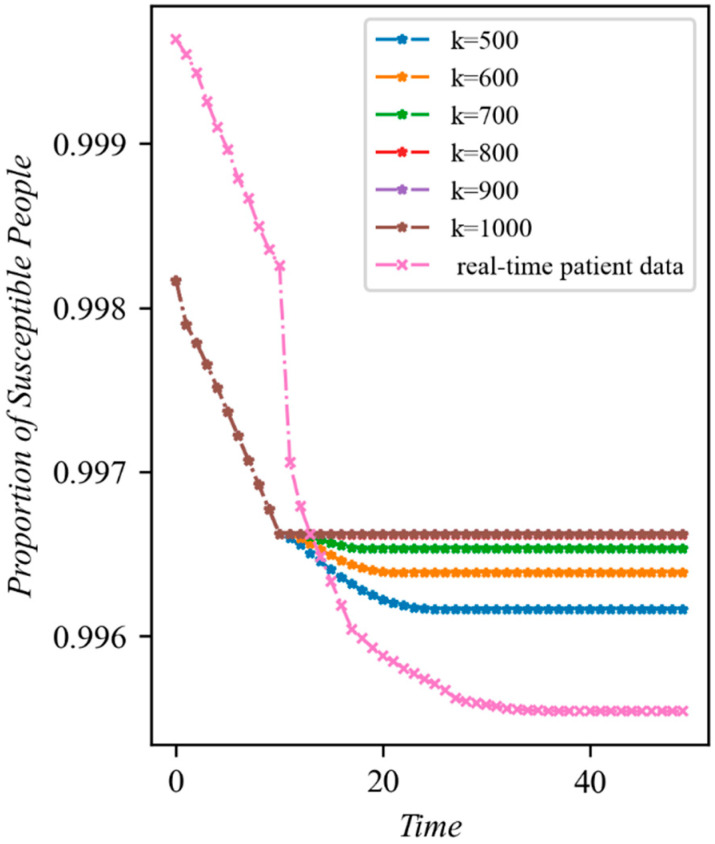
Changes in the number of susceptible individuals over time with different quantities of new medical resources (τ=0.95, η=10, date=10). The horizontal coordinates represent the time, and the vertical coordinates represent the proportion of susceptible population to the total population. The real-time data starts from 29 January 2020, which is 10 days before the first Fangcang shelter hospital began to operate.

We first tested the model’s ability to demonstrate the usefulness of allocating medical resources to Fangcang shelter hospitals to stop the epidemic by varying the number of infections under various Fangcang shelter constructions. The development of infections under various medical resource addition rates and using real-time patient data in Wuhan from 29 January is shown in Figure 4. Generally speaking, the share of the sensitive population that remains after the epidemic has stabilized increases as the number of new medical resources increases, and the tendency of susceptibility to reduce is greatly restrained following the closure of the Fangcang hospital. More specifically, the larger the proportion of the susceptible population is once the epidemic stabilizes, the more noticeable the change in the decreasing trend observed in the susceptible population is, and the sooner the epidemic reaches stability. Finally, the proportion of sensitive individuals in Time≤10 and K≥800 is essentially the same. The former is due to the fact that the model’s initial parameters are the same prior to the use of the Fangcang shelter hospital, which causes the model to evolve similarly. The latter is due to the fact that when medical supplies are very sufficient, all patients enter the Fangcang shelter hospital and the designated hospital, and there is no longer anyone in the community able to transmit infection, which prevents the susceptible population from changing. It is clear that setting up a Fangcang shelter hospital can significantly reduce the rate at which susceptible individuals get infected. If there are enough beds in Fangcang shelter hospitals, the spread of diseases can be promptly stopped even if there are not nearly enough of them to fully mitigate the sharp rise in the number of illnesses. This is consistent with earlier research [23] that produced comparable findings, highlighting the significance of Fangcang shelter hospitals with regard to outbreak prevention and control. In particular, when conduct simulations using data from the Wuhan epidemic in early 2020 and the real-time patient data, we can see that a similar trend to the simulation results is exhibited. This also shows the practical feasibility of the model in reality.

**Figure 5:**
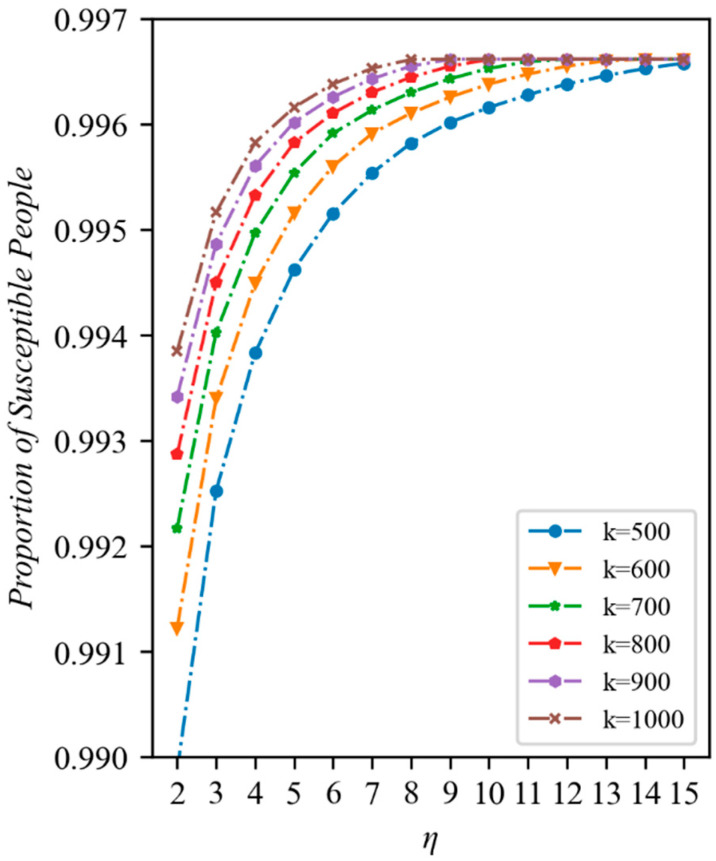
Number of susceptible individuals after stabilization with different quantities of new medical resources and conversion rates (τ=0.95, date=10). The horizontal coordinate represents the conversion rate of medical resources and the vertical coordinate represents the ratio of the susceptible population to the total population once the epidemic has stabilized.

We then immediately conducted an examination of how various conversion rates and allocation ratios contributed to the outbreak’s control. The role of increasing the conversion rate under various additional medical resources is shown in Figure 5. The proportion of susceptible population increases with the conversion rate under varied quantities of extra medical resources after the outbreak stabilizes, indicating that raising the conversion rate of medical resources can help to stop the outbreak from spreading. Scenarios with additional medical resources were able to benefit the highest level of susceptible population at a lower conversion rate and had a higher proportion of susceptible population at the same conversion rate, showing that the quantity of additional medical resources was crucial to controlling the epidemic’s spread. When the number of beds in the Fangcang shelter hospital are abundant and therefore redundant, all mild patients are admitted, and the epidemic no longer spreads. The different scenarios with varying quantities of additional health care resources, aside from k=500, eventually reached the same level of susceptibility.

It can be seen that with fixed resources, increasing the conversion rate can raise the maximum carrying capacity of Fangcang shelter hospitals, which is consistent with our prediction. Although there is theoretically no upper limit to the conversion rate, in practice, the work intensity and energy of health care workers are limited, and the conversion rate cannot be increased indefinitely. Increasing the conversion rate can have the effect of alleviating the lack of resources when resources are relatively scarce. If resources are available in sufficient quantities, excessively raising the conversion rate will not improve the preventative outcomes due to the fact that the Fangcang shelter hospital has enough space to accommodate all of the patients with a mild illness; indeed, adding more beds would only result in excess and increase the workload of the medical personnel.

**Figure 6:**
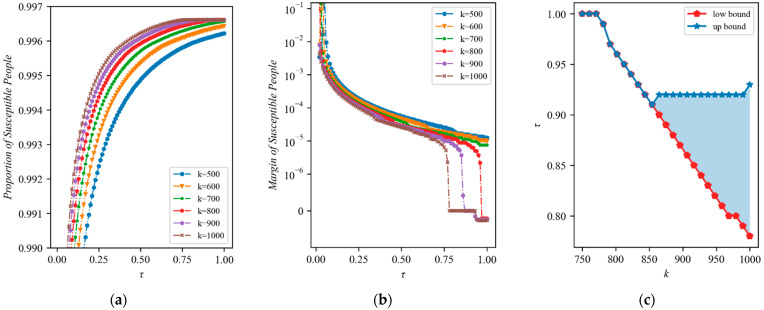
(**a**) Number of susceptible people after stabilization under different quantities of additional medical resources and allocation ratios (η=10, date=10); (**b**) Marginal changes in the number of susceptible individuals after stabilization as the allocation ratios increase cause different quantities of additional medical resources and allocation ratios (η=10, date=10 ); (**c**) The interval of the distribution ratio of the least number of infections under different conditions of additional medical resources (η=10, date=10 ). The shaded area represents the optimal allocation ratio, and the red and blue lines represent the upper and lower bounds of the optimal allocation ratio, respectively.

The effect of increasing the allocation ratio of new medical resources is shown in Figure 6a. Overall, as the allocation ratio rises, the proportion of susceptible populations keeps rising, showing that the epidemic can be prevented to a greater extent by allocating medical resources to Fangcang shelter hospitals. In particular, in each scenario, an increasing trend is observed, and the upward trend is accentuated by the availability of more medical resources. Increasing the allocation ratio might similarly prevent the spread of the outbreak. However, this hypothesis is made with the assumption that there are a finite number of resources available. In practice, it is still possible that seriously ill people might remain untreated in a period.

As the allocation ratio rises, Figure 6b shows the marginal amount of change in the susceptible population after the epidemic has stabilized. Overall, when the allocation ratio rises, the marginal change in the population that is vulnerable declines. Two trends in particular emerged in the marginal change. When the number of new resources is small, the marginal change is finally stable, and when the quantity of new medical resources is large, the marginal change further drops and enters a short-term platform; this continues to decline after the allocation ratio rises, indicating that the excessive allocation ratio at this time is not conducive to epidemic prevention. As a result, raising the allocation ratio can generally help to lessen the effects of epidemics, but this benefit is small and diminishes over time. It is important to note that the amount of change is negative when the allocation ratio is high and it is assumed that there are more sufficient resources; this indicates that, after a certain allocation ratio, continuing to increase the allocation ratio will actually result in an increase in the number of infections because too many severely ill patients cannot be treated due to a lack of medical resources in the designated hospitals. Therefore, having a higher proportion of Fangcang shelter hospital input does not make sense when there are more resources available.

The allocation ratio with the highest proportion of susceptible populations under scenarios with different quantities of total new medical resources is identified via the comparison of the proportion of susceptible populations. Figure 6c displays the best allocation ratios for new medical resources under various conditions. The optimal allocation ratio can be divided into three segments: at k≤770, the upper and lower bounds are equal and both equal to 1; at 770 to 850, the optimal allocation ratio gradually declines and the upper and lower bounds remain the same; and at k>850, the upper and lower bounds start to diverge. The lower bound maintains a linear downward trend, while the upper bound rises slightly and then plateaus.

**Figure 7:**
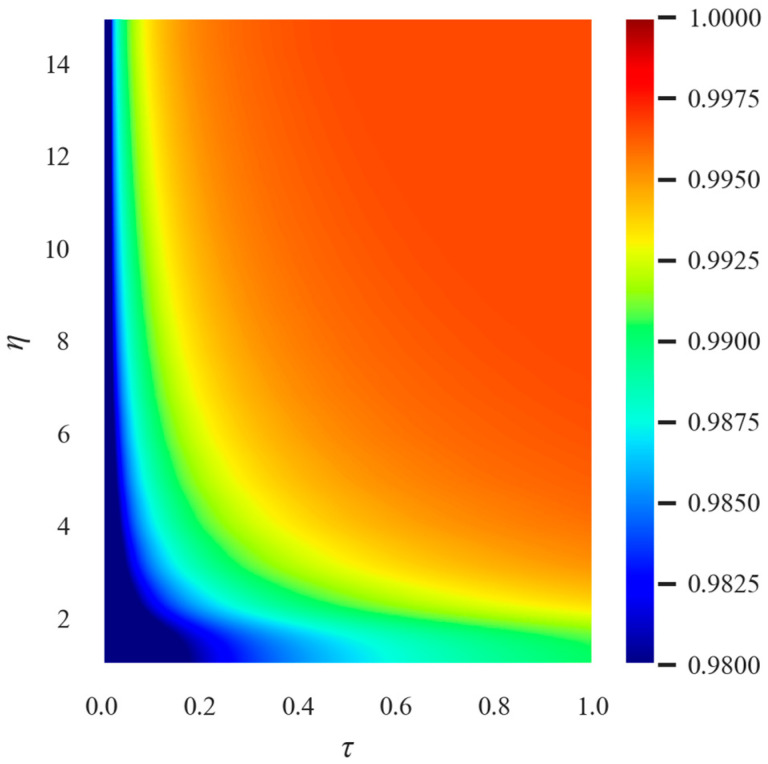
Remaining number of susceptible people with different allocation ratios and conversion rates under the scenario of fixed new medical resources. (k=1000, date=10). The horizontal axis represents the allocation ratio, the vertical axis represents the conversion rate, and the color represents the total number of susceptible people.

The combined impact of the conversion rate and allocation ratio on the efficiency of epidemic prevention was then simulated as shown in Figure 7. **s** depicts the epidemic’s spread under the scenario of fixed medical support with various allocation ratios and conversion rates. Overall, when the conversion rate and allocation ratio are both higher, the proportion of susceptible population is larger and epidemic prevention is more effective. With the same level of epidemic prevention, there is a specific inverse proportional relationship between the distribution ratio and conversion rate. If the conversion rate and distribution ratio reach a certain level, the proportion of the susceptible population no longer rises, and the epidemic prevention effect in this region is the same.

As can be seen, the upper bound of the allocation ratio does not change as the conversion rate increases. The lower bound of the allocation ratio under the optimal parameters decreases and exhibits a certain inverse relationship with the increase in the conversion rate, which is consistent with our prediction. The best epidemic prevention can only be achieved at a given resource quantity when the minimum combination of Fangcang shelter hospital and a designated number of hospital beds is met. To meet this demand, a minimum conversion rate needs to be attained in order to ensure that a sufficient number of Fangcang shelter hospital beds can be provided. As the conversion rate increases, the selected interval of the allocation ratio gradually increases, but the maximum cannot be exceeded.

We further confirmed the change in the number of deaths, with the lowest number of deaths occurring in the same parameter domain as when the number of infections was lowest, demonstrating that excessive medical resource consumption does not result in better epidemic prevention. The differences between the scenarios of absolutely insufficient resources (k=100) and absolutely adequate resources (k=10,000) were also further examined. The findings demonstrate that the best solution when resources are insufficient is to allocate all available resources to hospitals and increase the conversion rate to the highest level in order to stop the spread of the epidemic; however, when resources are sufficient, a lower conversion rate can produce the same epidemic prevention effect, and the upper bound is reached.

## 4. Discussion

A two-stage infectious disease model was constructed in this paper. The model takes into account the limited medical resources based on the conventional SEIR model, adds the maximum capacity of the designated hospitals and the Fangcang shelter hospitals, as well as various admission strategies that denote the two stages. In the first stage, all patients were admitted to the designated hospital, and in the second stage, patients were triaged; thus, mild patients entered the Fangcang shelter hospital first, and severe patients entered the designated hospital, and the transition between the two stages was marked by the operation of the Fangcang shelter hospital. Furthermore, we defined an allocation ratio in order to represent the method used to allocate healthcare resources, and also defined a medical resource conversion rate in order to demonstrate the advantages of Fangcang shelter hospital regarding their admission of minorly affected patients. Using these two parameters, we investigated the role of health care resource allocation strategies in stopping the spread of the epidemic.

In order to examine the effects of medical resource allocation on epidemic prevention and control, we analyzed the function of Fangcang shelter hospitals and their impact on epidemic prevention and control, as well as the influence of medical resource allocation. Our analysis demonstrates, firstly, that Fangcang shelter hospitals can successfully slow the spread of epidemics, which is consistent with previous research. Second, when medical resources are scarce, increasing the conversion rate and the proportion of input to Fangcang shelter hospitals can help stop the spread of the epidemic rapidly; however, when medical resources are abundant, excessively increasing either the conversion rate or the proportion of input to Fangcang shelter hospitals is detrimental to the overall prevention and control of epidemics. This may be explained by the fact that when medical resources are insufficient, putting resources into Fangcang shelter hospitals can minimize the potential transmission among the public by admitting as many minorly affected patients as possible; meanwhile, when there is a certain amount of spare medical resources, over-investing in Fangcang shelter hospitals can produce a wasteful excess of resources and designated hospitals require investment in order to save seriously affected patients.

## 5. Conclusions

This paper develops a two-stage model of infectious disease to examine the role of Fangcang shelter hospitals in outbreak prevention and control, and examines the impact of healthcare resource allocation on outbreak prevention and control. Our findings demonstrate that Fangcang shelter hospitals can effectively control the rapid spread of epidemics, which is consistent with previous research. Furthermore, we further discuss what the optimal medical resource allocation solutions are when medical resources are either limited or abundant. The results show that the optimal allocation ratio of resources between designated hospitals and Fangcang shelter hospitals varies with the amount of additional resources.

The results of our study’s analysis of allocation ratios and conversion rates may inform further developments in healthcare resourcing policy. Firstly, the early diagnosis and elimination of an epidemic are the cornerstones of prevention strategies. More medical resources are helpful during epidemic prevention and control if the disease has already spread to a given area, but excessive resource investment will lead to resource redundancy and ineffectiveness. Secondly, the government should allocate as many medical resources as possible to large-scale isolation measures, such as Fangcang shelter hospitals, if medical resources are insufficient. Meanwhile, it also needs to guarantee a certain number of beds for serious illnesses in designated hospitals. To prevent medical staff from becoming overwhelmed, the use of medical resources should be organized intelligently. However, overwhelmed medical staff will not result in a greater contribution to the prevention of epidemics. Finally, in order to achieve the best epidemic prevention and control objectives, the government’s medical resource allocation policy might consider both minorly and seriously affected patients, isolation admission and medical treatment, and flexible disposal within a reasonable interval, combined with the actual situation.

Our work is not without limitations. Firstly, we did not consider the social and economic costs in our model. Indeed, from an economic perspective, future research could also examine the long-term effects of short-term investments in Fangcang shelter hospitals and designated hospitals. This is because allocating medical resources to either the designated hospitals or Fangcang shelter hospitals requires the consideration of whether to renovate the existing site or build a new one, as well as the long-term future operations. Secondly, the parameters of our model are static, which may not be the case in the real world. Although we set key parameters using some realistic scenarios, these parameters remain constant. Future research could focus on the dynamics of infectious disease models with multiple thresholds. Further research using realistic and feasible optimization scenarios and employing operational research methods could contribute from an optimization perspective.

Finally, our paper offers guidelines regarding the allocation of resources in such Fangcang shelter hospitals. The effects of the rational governmental allocation of health care resources vary during epidemics. Combined with reality, healthcare resources are flexibly allocated and deployed in order to adjust and therefore maximize the benefits of these measures. Our work exploring the impact of Fangcang shelter hospitals on epidemic prevention and control has improved our understanding of the impact of healthcare resource allocation on infectious disease prevention and control, and will inform future disease prevention and control responses.

## Data Availability

The data supporting our results comes from our designed procedure. The procedure code and the results are included within the Supplementary Material files.

## Supplementary Materials

The following are available online at https://www.mdpi.com/article/10.3390/ijerph20105802/s1, Table S1: Operational definitions of each parameter. The folder “code” contains the source code for the model proposed in the paper.
