# Supplementary material for: The Effect of Fangcang Shelter Hospitals under Resource Constraints on the Spread of Epidemics
Source: Int J Environ Res Public Health. 2023 May 12;20(10):5802. doi: 10.3390/ijerph20105802 (PMC10217850; doi:10.3390/ijerph20105802)
Supplement: Supplementary file 1 [file ijerph-20-05802-s001.zip › Supplementary Material.pdf]

Table S1: Operational definitions of each parameter.
The folder “code” contains the source code for the model proposed in the paper.

Table S1. Operational definitions of each parameter

Parameter Type	Parameter Name	Parameter Meaning
Disease parameters	β	The average number of persons a susceptible person can be exposed to and infected per unit of time without being confined is indicated by the pace at which they become infected.
	ω	Incidence of latency patients, the inverse of which is the average latency period of onset.
	δ	Severe sickness rate, which shows the risk of a mild patient developing into a severe condition per unit of time without treatment and shows the possibility of a mild patient's condition deteriorating at home.
	γ	The probability that a patient with a mild disease will recover per unit of time without treatment, or the rate of recovery of a patient with a mild illness, measures the possibility that a patient with a mild sickness will recover on his own at home.
	θ	The likelihood that a severely ill patient would pass away without treatment per unit of time is indicated by their mortality rate.
Resource Parameters	$date$	The date of the Fangcang Hospital's founding designates the interval between the time the government learned of the confirmed case and began taking preventive measures and the time the Fangcang started taking in patients with mild ailments.
	T	The first phase's maximum number of beds in designated hospitals, which represents the number of beds in designated hospitals prior to the construction of the party cabin, shows the local community's reserve of medical resources.
	T'	The total of the beds in the first stage plus the new beds makes up the maximum number of beds in the second stage of the fixed hospital, which represents the number of beds in the fixed hospital following the installation of the Fangcang module.
	T_C	The maximum number of beds in the second phase of the Fangcang shelter hospital serves as a proxy donates the number of beds in the Fangcang following its establishment.
	k	Total extra medical resources, which represents how many authorized hospital beds can be converted from assisted medical resources, is a reflection of how many medical resources have been recruited from nearby places.
	τ	The medical resource allocation ratio donates the input side cabin hospitals shows the proportion of new medical resources to all resources.
	η	Medical resource conversion rate, which shows the proportion of beds in designated hospitals with the similar medical resources to beds in Fangcang shelter hospitals.
Medical Parameters	α_1	The multiple of the increase in the chance of patients being cured after medical intervention is shown by the coefficient of change in the rate of cure of patients with mild illnesses following medical intervention.
	α_2	Coefficient of change in the rate of recovery of severely ill patients without access to medical care, showing the multiple of the decline in the likelihood that critically ill individuals will heal on their own.
	α_3	The successive reductions in the likelihood of severely sick patients recovering following medical intervention are shown by the

	coefficient of change in the rate of healing donates those patients.
α_4	An indicator of the numerous mortalities decreases in critically sick individuals following medical intervention is the coefficient of change in mortality.
α_5	The multiple decreases in the likelihood of an exacerbation in patients with small diseases after medical access are indicated by the coefficient of change in the rate of serious illness after medical intervention.
